# Pathophysiology of Nociception and Rare Genetic Disorders with Increased Pain Threshold or Pain Insensitivity

**DOI:** 10.3390/pathophysiology29030035

**Published:** 2022-08-02

**Authors:** Marco Cascella, Maria Rosaria Muzio, Federica Monaco, Davide Nocerino, Alessandro Ottaiano, Francesco Perri, Massimo Antonio Innamorato

**Affiliations:** 1 Division of Anesthesia, Istituto Nazionale Tumori IRCCS—Fondazione G. Pascale, 80131 Naples, Italy; monacofederica@gmail.com (F.M.); dr.nocerino@gmail.com (D.N.); 2 Division of Infantile Neuropsychiatry, UOMI-Maternal and Infant Health, ASL NA3/Sud, 80059 Naples, Italy; maramuzio@yahoo.it; 3 SSD Innovative Therapies for Abdominal Metastases, Istituto Nazionale Tumori IRCCS—Fondazione G. Pascale, 80130 Naples, Italy; a.ottaiano@istitutotumori.na.it; 4 Medical and Experimental Head and Neck Oncology Unit, Istituto Nazionale Tumori IRCCS—Fondazione G. Pascale, 80131 Naples, Italy; f.perri@istitutotumori.na.it; 5 Department of Neuroscience, Pain Unit, Santa Maria delle Croci Hospital, AUSL Romagna, Viale Vincenzo Randi 5, 48121 Ravenna, Italy; massimo.innamorato@auslromagna.it

**Keywords:** nociception, pain insensitivity, nociceptors, hereditary sensory and autonomic neuropathies, Angelman syndrome, Prader Willy syndrome, Chromosome 15q duplication syndrome, Chromosome 4 interstitial deletion, cancer

## Abstract

Pain and nociception are different phenomena. Nociception is the result of complex activity in sensory pathways. On the other hand, pain is the effect of interactions between nociceptive processes, and cognition, emotions, as well as the social context of the individual. Alterations in the nociceptive route can have different genesis and affect the entire sensorial process. Genetic problems in nociception, clinically characterized by reduced or absent pain sensitivity, compose an important chapter within pain medicine. This chapter encompasses a wide range of very rare diseases. Several genes have been identified. These genes encode the Nav channels 1.7 and 1.9 (*SCN9A*, and *SCN11A* genes, respectively), *NGFβ* and its receptor tyrosine receptor kinase A, as well as the transcription factor PRDM12, and autophagy controllers (*TECPR2*). Monogenic disorders provoke hereditary sensory and autonomic neuropathies. Their clinical pictures are extremely variable, and a precise classification has yet to be established. Additionally, pain insensitivity is described in diverse numerical and structural chromosomal abnormalities, such as Angelman syndrome, Prader Willy syndrome, Chromosome 15q duplication syndrome, and Chromosome 4 interstitial deletion. Studying these conditions could be a practical strategy to better understand the mechanisms of nociception and investigate potential therapeutic targets against pain.

## 1. Introduction

According to the International Association for the Study of Pain (IASP), pain is “An unpleasant sensory and emotional experience associated with, or resembling that associated with, actual or potential tissue damage” [1]. This definition, and the set of proposed notes, have completely changed the semantic and clinical approach to one of the most frequent symptoms. Notably, a survey conducted by The National Center for Health Statistics, in 2019, demonstrated that about one in five adults in the United States suffered from chronic pain [2]. Among the descriptive notes, the IASP underlines that “Pain is always a personal experience that is influenced to varying degrees by biological, psychological, and social factors” and, importantly, “Pain and nociception are different phenomena”. Briefly, nociception is the result of the complex activity in sensory neurons. On the other hand, pain is the effect of interactions between nociceptive phenomena, experiences, emotions, social context, and other elements that are sometimes difficult to identify and dissect. 

Through the activation of peripheral terminals, conduction of transduced stimuli to the medulla, centripetal pathways, and modulations occurring at different levels, nociception configures the structural part within the pain process. It works like a domino effect in which one event causes a series of related events, one after another. Nociception can be defined as the reception of signals from the CNS for the activation of peripheral nociceptors. It includes all neurophysiological and neurochemical events related to pain sensitivity. Although not all nociceptive stimuli are necessarily followed by signal transduction (and pain), the proper ones trigger the domino effect of nociception. Therefore, nociception is responsible for the sensory-discriminative component of pain. For example, nociceptors inform us about the start, intensity (quality), and end of the stimulus. The other components, including the affective, emotional, cognitive (e.g., the meaning of the pain experience in relation to the health status), autonomic, and motor components, enrich sensation with contents and, finally, shape the whole pain experience.

The pathological processes of a different nature can alter the nociception process. Trauma, inflammation, neoplasms, and metabolic alterations, as well as chemical, physical, and biological agents, can interfere with this intricate mechanism. The clinical effect is variable and depends on the type of lesion. At the same time, endogenous substances, drugs, and physical agents impact the system to modulate nociception and produce analgesic effects. Furthermore, twin studies demonstrated hereditary components in several chronic pain syndromes, such as irritable bowel syndrome, musculoskeletal pain, pelvic pain, and dry eye disease, although the role of genetic and environmental factors should be better explained [3]. On the other hand, a heterogeneous group of genetic disorders is phenotypically characterized by increased pain threshold or pain insensitivity. Congenital insensitivity to pain (CIP) and hereditary sensory and autonomic neuropathy (HSAN) are examples of Mendelian pain loss conditions [4], but several numeric and structural chromosomal abnormalities are also described. Clinical features encompass pain alterations combined or not with a variety of neurological manifestations, as well as symptoms affecting different organs and systems.

Despite the enormous impact of chronic pain issues on both patients and healthcare systems, progress in the development of new therapeutic agents is limited. Commonly, pharmacological strategies are aimed at optimizing the drugs and tools available to clinicians (e.g., through multimodal and tailored approaches). Nevertheless, new drugs that can guarantee an effective and safe action profile are needed.

In this narrative review, the authors describe the main features of nociceptors and discuss genetic disorders characterized by increased pain threshold or pain insensitivity. Finally, research perspectives in preclinical and clinical research are reported.

## 2. Materials and Methods

This narrative review was performed by collecting clinical trials, basic research, and reviews on nociception and rare genetic disorders characterized by reduced or absent pain sensitivity. Articles published in peer-reviewed scientific journals were included. Articles were excluded if they were not written in the English language.

### 2.1. Search Strategy

We researched the PubMed database using keywords or combinations of keywords.

“Nociception”, “Pain insensitivity”, “Nociceptors”, “Hereditary sensory and autonomic neuropathies”, “Angelman syndrome”, “Prader Willy syndrome”, “Chromosome 15q duplication syndrome”, “Chromosome 4 interstitial deletion”. Articles published between 1 January 2005 and 30 March 2022 were selected. We further screened articles from peer-reviewed scientific journals that were written in English.

### 2.2. Study Selection

We screened for relevant articles and selected articles for further reading based on the title and abstract. We read the articles that potentially fit our topic and all included articles were carefully discussed in the present review. This narrative review does not need ethical approval, because no human/patient data were used.

## 3. Results

**Figure 1:**
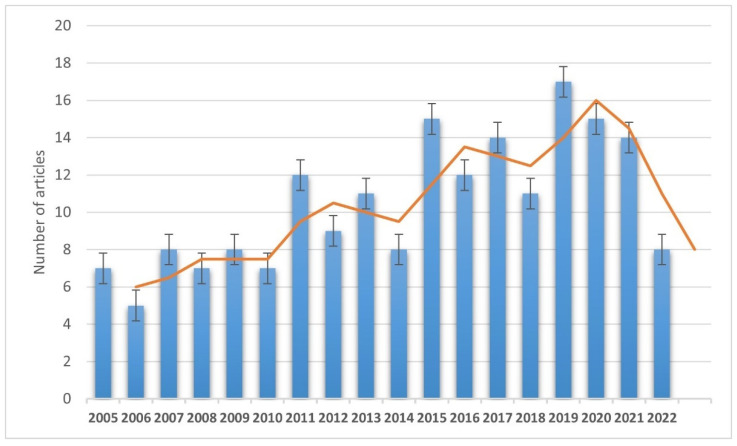
Annual trend of selected and discussed publications (from 1 January 2005 to 30 March 2022).

Four hundred and fifty articles were included from the initial search, and 189 articles were selected and discussed in the review in the end (Figure 1).

### 3.1. Features of Nociceptors

The starting point of nociception is the activation of specialized structures, namely the nociceptors [5]. They are relatively unspecialized nerve cell endings that initiate the process of pain sensation. These terminals arise from cell bodies in dorsal root ganglia (DRG) or in the trigeminal ganglion; from the cell body, one axonal process reaches the periphery (nociceptor), and the other extension projects into the spinal cord or brainstem.

The viscera and part of the gastrointestinal system also have vagal afferents innervation and their cell bodies are located in nodose ganglia (NG), but it remains unclear if neurons in NG convey nociceptive sensation.

The study of nociceptors is a very important research topic, especially for potential therapeutic implications [6]. The function of these structures is conditioned by an indefinite number of factors, not only pathological in nature. For example, preclinical studies have shown important age and sex differences for this purpose [7]. Moreover, although the main role of nociceptors is sensory discrimination, they can also affect other processes. For example, nociceptors activation can increase capillary permeability and blood flow, producing neurogenic inflammation [8].

As a general rule, nociceptors are activated by several nociceptive stimuli, including inflammatory mediators, such as interleukins, prostaglandins, and cytokines, as well as neuropathic (due to damage), and nociceptive (thermal, chemical, and mechanical) stimuli. This distinction is purely academic because the processes often overlap. For instance, although tissue damage can usually trigger an inflammatory response, nerve damage and/or activation of nociceptors that respond to physical insults can be related.

Since nociceptors are “free nerve ending” structures, they are included among the classes of somatic sensory receptors and classified on the properties of the axons associated with them. In summary, axons conveying information about pain are mostly Aδ myelinated axons or C fibers of unmyelinated axons. The former type (Aδ) includes mechanosensitive and mechanothermal nociceptors, which exhibit fast conduction (about 20 m/s); on the contrary, the C fibers are polymodal nociceptors (mechanosensitive), conducting at velocities generally less than 2 m/s (slow pain pathway). Not all C-fibers are fully polymodal. A subgroup is formed by the C-mechanoinsensitive afferents. They are unresponsive to mechanical stimuli, although activated by thermal responses (at >48 °C) and various chemical algogens. Another group of thickly myelinated nociceptors is the Aβ type. These mechanosensors have conduction velocities like those of Aδ fibers and are activated by high-threshold stimuli [9]. The mechanically insensitive or silent nociceptors are unresponsive to all modalities but respond in the inflammatory state [10]. (Table 1).

Alterations at certain stages of the sensorial route can affect the entire nociception process. These changes can involve the first phase of nociception, namely the signal transduction events within the nociceptor. Nociceptive transduction is the process by which stimuli are converted to electrical signals that can be carried (via transmission) to the central nervous system (CNS).

Clinically, the effects produced by problems in this fundamental step of the nociceptive pathway vary from hyperalgesia phenomena to increased pain tolerance until pain insensitivity. Although multiple causes can provoke these alterations, most of these occur due to an acquired process. In particular, nociception modifications can manifest in degenerative processes (e.g., Parkinson’s disease) [11] or for instance because of a sustained suprathreshold mechanical stimulation [12]. Nevertheless, peripheral sensitization with increased nociceptive response to mechanical and chemical stimulation is among the underlying mechanisms of chronic pain. The immune system has an important role in nociception modulation. During inflammation, cytokines, and chemokines can reduce the nociceptor thresholds and through an increased sensitivity to stimuli, inflammatory pain hypersensitivity can develop [13]. Acquired processes can also affect the tissue density of fibers, specifically the intraepidermal nerve fiber density (IENFD). Remarkably, decreased IENFD is a marker of neuropathy and has been shown in several painful clinical manifestations, such as diabetic polyneuropathy [14], complex regional pain syndrome [15], and Guillain–Barré syndrome [16].

Nociceptors are usually categorized into peptidergic and non-peptidergic subgroup families. Human nociceptors likely have a peptidergic phenotype [5]. The latter nociceptors express calcitonin gene-related peptide (CGRP) and/or substance P (SP). They work through the release of neuropeptides from their peripheral terminals in a calcium influx dependent manner (pain modulators). On the other hand, non-peptidergic populations bind isolectin B4 (IB4) and/or express purinoceptor P2X3 (activated by extracellular ATP) [17].

**Figure 2:**
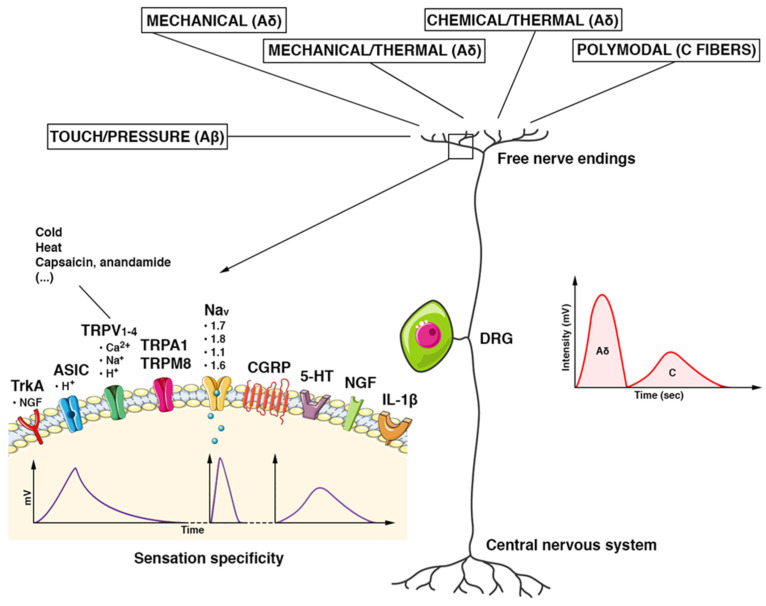
Schematic features of the DRG nociceptor. The pseudounipolar neuron has its cell body located in the dorsal root ganglion (DRG). Once the stimuli reach the terminal receptor, an ionic current is generated. It provokes a gradual potential that modulates the discharge of action potentials in the afferent nerve fibers directed to the CNS. Each receptor generates an ionic current with its characteristic voltage response (intensity (mV) and duration (Time)) and determines the specificity of the sensation. Stimuli are carried by multiple types of fibers. Tactile/pressure stimuli are conducted by Aβ fibers, mechanical/thermal/chemical stimuli by Aδ fibers, and polymodal stimuli by C fibers. Aδ fibers are myelinated, fast-conducting fibers. They conduct the first part of the pain stimulus, which is the most defined, acute, and superficial one (a spike of intensity in a shorter time). The non-myelinated C fibers are slow-conducting and convey the second part of a pain stimulus in a more lasting, profound, and less defined way (reduced in intensity and longer in the time). Abbreviations. TrkA: tropomyosin kinase A receptor; ASIC: acid-sensitive ion channel; TRPV1–4: transient receptor potential cation channel subfamily V; TRPA1: potential cation channel of the transient receptor, subfamily A, member 1; TRPM8: member of the M (melastatin) subfamily of the potential cation channel of transient receptor 8; Nav (1.1–1.8): voltage-gated sodium channels; CGRP: peptide related to the calcitonin gene; 5-HT: 5-hydroxytryptamine; NGF: nerve growth factor; IL-1β: Interleukin 1 beta.

In each nociceptor subgroup, there is a different expression of structural/functional elements, namely nociceptive transduction channels (Figure 2). An important family is formed by the transient receptor potential (TRP) cation channels. Although most TRP channels transport calcium, other cations, such as sodium, magnesium, or iron, can be transported. TRP channels are ubiquitously distributed in the body. Several hereditary diseases caused by defects in TRP genes, such as polycystic kidney disease and mucolipidosis type IV, have been described [18]. Some of the so-called thermosensitive TRP channels play a key role in nociception [19]. The TRP vanilloid 1 (TRPV1) channel is mostly localized at the peptidergic terminal [20]. Another ion channel that is activated by environmental irritants is the TRP ankyrin-repeat 1 (TRPA1) channel. It is found in the non-peptidergic human DRG neurons, mostly in association with TRPV1 expression [21]. Furthermore, the TrkA (NTRK1 gene) is exclusively expressed in the peptidergic DRG subpopulation. To note, it exhibits a high affinity for nerve growth factor (NGF) receptors [22,23]. NGF belongs to the neurotrophin family and plays a fundamental role in the differentiation and survival of neurons (including DRG) during embryogenesis. In adults, NGF influences the nociceptive neuronal activity (pain modulators). The upregulation of neurotrophins and receptors expression, as well as the increased DRG excitability, are encompassed among the multiple pathogenic mechanisms of chronic pain conditions, such as postherpetic neuralgia [24].

In both peptidergic and non-peptidergic subgroups, voltage-gated channels have an important role in nociception. For example, the voltage-gated sodium (Nav) channels 1.7, 1.8, and 1.9 are somewhat specifically expressed in DRG nociceptors and they can be used for targeted pain therapy. 

The composition of the nociceptors (e.g., channels, modulators) is truly complex and, within certain limits, is subjected to continuous reworking. It depends on the innervated territory, type of stimulus (duration, intensity), and concomitance of factors that can alter their functioning (e.g., drugs, pathologies). Furthermore, recent studies have shown that the gene expression of DRGs is much more complicated than expected [25]. Among the multiple examples of implicated genes in pain and other processes, there are CCKAR, OSM, IL31RA, and CHRNA9 genes. The latter encodes the nicotinic alpha 9 receptor and can be useful as a target against chemotherapy-induced neuropathy (CIPN) [26,27].

### 3.2. Genetic Disorders Featuring Increased Pain Threshold or Pain Insensitivity

Genetic problems in the signal transduction of nociceptive stimuli configure an important and underestimated chapter in pain medicine. These disorders can involve ion channels (channelopathies) or other key elements of transduction pathways, such as modulators. Alterations in painful sensitivity are found in multiple clinical pictures. For narrative purposes, hereditary sensory and autonomic neuropathies (HSANs) [4], and numeric and structural chromosomal abnormalities are reported. In the former group (Mendelian disorders), nociception is altered due to a mutation in an ion channel (channelopathies), structural neuronal elements, nociception modulators, or transcription factors. In complex genetic alterations, although clinical findings are suggestive of a reduced/absent sensitivity to pain, the underlying pathogenetic mechanisms are often difficult to identify. Moreover, this approach is limited by continuous updates because of the discovery of new mutations and/or the more precise identification of genotype–phenotype correlations. As Lischka et al. [4] recently highlighted, a precise terminology is still lacking.

#### 3.2.1. Hereditary Sensory and Autonomic Neuropathies (HSANs)

HSANs encompasses a variegated group of inherited neuropathies, displaying sensory and autonomic alterations. In some forms, a congenital sensory deficit occurs, and these subtypes are also indicated as congenital insensitivity to pain syndromes.

The classification of HSANs is based on the underlying genetic mutation, inheritance pattern, and clinical characteristics. According to Schwartzlow et al. [28], eight phenotypically HSAN types can be described (HSAN-I to HSAN-VIII) and in each type, several subtypes have been identified. Another form (HSAN-IX) is due to a mutation of the TECPR2 gene located on Chromosome 14 (Table 2).

The diagnosis of HSAN is based upon clinical evaluations, a detailed patient/family history, and a variety of diagnostic tests (e.g., sequencing, electromyography).

##### HSAN-I Group

HSAN-I group is the most common type of HSANs. The diseases of this group have an autosomal dominant pattern of inheritance and different genetic mutations. They are mostly involved in the SPTLC1 gene (HSAN-IA) and lead to reduced activity of serine palmitoyltransferase, which is a key enzyme in a pivotal step of the sphingolipid synthesis. Signs and symptoms appear in the second to fourth decades of life and consist of pupillary abnormalities, loss of corneal reflex and abrasions, deafness, restless legs, cramps, absent reflexes, Charcot joints, and a propensity to pick up painless injuries of the tongue and limbs. The clinical picture can be complicated with the occurrence of osteomyelitis and sepsis [29,30].

In this variant, the sensory disturbances of nociception are not linked to a channel or receptor defect, but to a membrane alteration with the accumulation of neurotoxins that impair the signal transduction. Other mutations have been reported. One of these affects the SPTLC2 gene (HSAN-IC) and produces an HSAN-IA-like phenotype [31]. Moreover, mutations at the ATL1 gene (HSAN-ID) with an altered synthesis of axonal proteins, as well as involving the DNMT1 gene (HSAN-IE) coding a DNA methylation protein [32] and ATL3 gene (HSAN-IF) [33] have been also described.

##### HSAN-II

This type has an autosomal recessive inheritance pattern. Four mutations have been reported. They concern the WNK1, FAM134B, KIF1A, and SCN9A genes, each one associated with a different subtype designated A through D [34]. Notably, WNK1 is engaged in organizing sodium and chloride ions fluxes and cell membrane excitability. Furthermore, it coordinates the expression of TRPV4, which is an important cation channel involved in nociception. This mutation leads to the HSAN-IIA phenotype featuring profound loss of pressure, proprioception, vibration sensation, areflexia, decreased corneal reflexes, diffuse hypotonia, and autonomic problems (episodic hyperhidrosis) [34]. Recessive mutations in KIF1A, encoding for kinesin proteins (axonal transport of synaptic vesicles), can be the cause of HSAN-II disorders featuring complex neurological pictures.

HSAN-IIB and HSAN-IIC are not associated with increased pain threshold or pain insensitivity. HSAN-IID subtype is also termed congenital insensitivity to pain type 1 (CIP1). It is due to a loss-of-function mutation of SCN9A, which produces an impairment of Nav1.7, a voltage-gated sodium channel found in nociceptive networks and olfactory nerves. Consequently, this type is characterized by pain insensitivity and hyposmia [35]. Many mutations of SCN9A and, consequently, Nav1.7 dysfunctions have been described. They do not necessarily induce reduced pain sensitivity [36]. Despite loss-of-function Nav1.7 mutants leading to congenital pain insensitivity, gain-of-function mutations cause the primary erythermalgia and paroxysmal extreme pain disorder. Primary erythermalgia is characterized by a lowered threshold for activation and slowed deactivation. These alterations produce pain attacks and erythema (particularly in the hands and feet) triggered by mild warming stimuli. Moreover, paroxysmal extreme pain disorder (previously known as familial rectal pain) is featured by paroxysmal attacks of visceral pain involving the lower body (e.g., rectum and genitalia) or eye pain. The flushing of the affected site and other autonomic disturbances can occur.

##### HSAN-III

This type is also known as Riley–Day syndrome or familial dysautonomia. It is a neurodevelopmental genetic autosomal recessive disorder due to different mutations of the IKBKAP or ELP1 gene (9q31.3). This gene encodes for the elongator complex protein 1 (ELP1), which is a scaffold protein for the transcription of neural key proteins and a regulator for different kinases involved in proinflammatory signaling [37]. The pathological findings demonstrate diffuse alterations, affecting myelinated terminals (usually small nerve fibers), DRGs, spinal cord lateral root entry zones, Lissauer’s tracts, and parasympathetic afferents.

Clinically, familial dysautonomia often presents at birth with progressive onset and course; it manifests with pain insensitivity or reduced pain sensitivity, impaired temperature perception, motor incoordination, hypotonia, and feeding difficulties. Dysautonomia is characterized by increased secretions, gastroesophageal reflux, vomiting crises, orthostatic hypotension and dysautonomic crisis (e.g., hypertension crises), loss of temperature control, and alacrima with subsequent corneal ulceration [28].

##### HSAN-IV

Among the genetic disorders involving mutations in genes encoding receptors or modulators expressing pain insensitivity there is the HSAN-IV. This condition is also referred to as congenital insensitivity to pain with anhidrosis (CIPA). It is an extremely rare syndrome because a few hundred cases have been described and the estimated incidence is approximately 1 in 125 million newborns [38]. In genetics, CIPA is an autosomal recessive disease with a loss-of-function mutation in the TRKA gene encoding the high-affinity tyrosine kinase receptor NTRK1 for NGF. This gene is located on chromosome 1 (1q21–q22) and several mutations have been reported. Moreover, a non-Mendelian inheritance characterized by uniparental disomy of chromosome 1 can occur [39]. 

The clinical presentation is characterized by three basic signs including global insensitivity to pain, anhidrosis, and intellectual disability [38]. In addition to pain problems, the sensorial impairment often includes an inability to feel temperature and often leads to repeated severe injuries. Other possible signs and symptoms are bone lesions, facial alterations, microcephaly, mandibular osteolysis, dental caries and premature tooth loss, recurrent soft tissue and bone infections, urine and fecal incontinence, and growth disturbances, as well as recurrent fevers, anhidrosis, self-mutilating behaviors, and hypotonia. The electrophysiologic investigations demonstrated absent lower limb sensory nerve action potentials in about 50% of patients and the biopsy can find the absence of unmyelinated fibers (15%) [40]. Symptoms are usually found at birth or during infancy. The therapy is symptomatic. Regarding bone lesions, Pérez-López et al. [41] proposed an early surgical treatment for long bone fractures and bisphosphonates use to address osteoporosis. Because of the complexity of the clinical pictures, a multi-professional approach modulated on the patient’s needs must guarantee the possibility of improving the quality of life of these patients.

##### HSAN-V

A specific mutation in the NGFβ gene (chromosome 1p13.2) causes the autosomal recessive HSAN-V disease. It is a very rare condition as only a few people with the condition have been identified. Concerning the phenotypic spectrum, Carvalho et al. [42] studied a family with five children who suffered from a congenital inability to feel pain, anhidrosis, abnormal temperature sensing, mild intellectual disability, and an immune deficiency. Other authors described a family with a homozygous NGF mutation and presented with congenital insensibility to pain, lack of temperature sensing, but normal sweating, immunity, and cognitive abilities. The biopsy demonstrated a reduced number of C-fibers [43].

The NGFβ gene binds with the TRKA gene (HSAN-IV) and is also involved with signaling apoptosis of nociceptive sensory neurons. Consequently, the HSAN-V phenotype can encompass HSAN-IV characteristics. Probably, a spectrum of phenotypes caused by changes in the NGF/TRKA signaling pathway can be postulated.

##### HSAN Type VI

This type was described, in 2012, by Edvardson et al. [42] in a single family of three Ashkenazi children with severe dysautonomic symptoms, distal contractures, severe psychomotor delays, pain insensitivity, and premature death. The underlined mutation involves the dystonin (DST) gene encoding dystonin. It is a member of the plakin family of proteins that bridge the cytoskeletal filament networks. Several isoforms, including neuronal (3), muscle (3), and epithelial (1), have been detected. Since the children were affected by a frameshift mutation starting at Glu4995, loss of a functional domain common to all major neuronal dystonin isoforms was postulated [44].

After the first report, Manganelli et al. [45] published the features of three affected Italian brothers with mutations of the neuronal isoform-a2 of DST. The authors described clinical pictures characterized by severely impaired pain sensitivity, distal ulcerations, amputations of fingers or toes, and joint deformities, as well as autonomic disturbances, including reduced sweating, heat intolerance, absent light reflexes, chronic diarrhea, and erectile dysfunction. Neurodevelopmental delays were not observed. The skin biopsy showed loss of Meissner corpuscles, myelinated fibers, and epidermal nerve fibers.

Based on the study of these two families, this very rare autosomal recessive defect is clinically characterized by serious autonomic dysfunctions and reduced/absent pain sensitivity. The clinical picture may vary according to the type of gene mutation and the protein isoform affected. In particular, the milder phenotype seems to be associated with mutations of the neuronal isoform-a2 of DST.

##### HSAN Type VII

In 2013, Leipold et al. [46] identified a specific de novo heterozygous gain-of-function mutation in the SCN11A gene (L811P domain) in two unrelated patients with a congenital inability to experience pain and frequent tissue damages. It was also indicated as CIP2 to differentiate it from CIP (or HSAN-IID) due to a loss of function SCN9A mutation. On the contrary, in this congenital pain disorder, SCN11A mutations are associated with a gain of function with the sustained depolarization of nociceptors that obstruct the generation of action potentials [47]. The SCN11A is preferentially expressed in DRG and trigeminal ganglia in which it encodes the NaV1.9 channel. The role of the Nav1.9 channel in pain mechanisms is still under investigation and its experimental deletion (Nav1.9-null) can diminish the thermal pain hypersensitivity in knock-out mice [46]. Nevertheless, it plays a key role in the generation of heat and mechanical pain hypersensitivity, both in subacute and chronic inflammatory pain models [47]. Moreover, this channel is also an important regulator of afferent sensitivity in visceral pain pathways to mechanical and inflammatory stimuli. These findings suggest that the Nav1.9 channel may represent a suitable pharmacological target for inflammatory and visceral pain. 

In this rare disorder, the phenotype is characterized by decreased pain and temperature sensations, hyperhidrosis, significant pruritis, self-mutilation, delayed healing, Charcot joints, scoliosis, hypotonia, delayed motor development, and intestinal dysmotility and diarrhea. A milder phenotype was described by Woods et al. [48]. The patient presented with abdominal and urinary discomfort with chronic constipation, mild hypotonia and muscle weakness, and an inability to feel peripheral pain, although intelligence was normal. In another case report, the patient suffered from severe abdominal, perianal, or rectal acute episodic pain during defecation or urination [49].

##### HSAN Type VIII

This disorder is also indicated as CIP3 since it presents at birth or early childhood with loss of pain and temperature sensations, and painless injuries. The syndrome is caused by mutations in the PRDM12 gene and is inherited in an autosomal recessive pattern. This gene encodes for the transcription factor PR domain containing member 12 (PRDM12). It is a member of a family of transcriptional regulators (epigenetic regulators) that participate in the control of vertebrate neurogenesis. Chen et al. [50] showed that PRDM12 plays a critical role in pain perception. Clinically, the sensory deficit is difficult to standardize in precise patterns and patients do not experience global pain insensitivity. For example, in their case series, Zhang et al. [51] described a patient with complete pain insensitivity in the left leg, but partial in the right. The clinical picture can include self-mutilation, dental trauma, osteomyelitis, anhidrosis, hyperpyrexia, absent corneal reflexes, and alacrima [50]. The phenotype is very similar to that of HSAN type IV and HSAN type V, but usually patients have normal intellectual abilities. In the literature, only a few cases have been described [51].

##### HSAN Type IX

The syndrome was previously indicated as spastic paraplegia [52]. It is due to a homozygous or compound heterozygous mutation in the TECPR2 gene (14q32.31) with impairment of the intracellular autophagy pathway. Clinically, this rare and severe syndrome is characterized by global developmental delay and intellectual disability, dysmorphic features (e.g., brachycephaly, microcephaly), hypotonia, dysarthria, hyporeflexia, central apnea, ataxic (Biot’s) breathing [53], and autonomic dysfunction (gait ataxia). Patients can be affected by peripheral neuropathy and decreased sensitivity to pain [54].

#### 3.2.2. Numeric and Structural Chromosomal Abnormalities

Aneuploidies and structural chromosomal abnormalities, such as terminal or interstitial deletions, inversions, translocations, or duplications, occur in about 0.6% of live births [55]. They often manifest as dysmorphism, malformations, and/or developmental disabilities. Alterations in pain sensitivity can be encompassed among the clinical features (Table 3).

##### Angelman Syndrome

Angelman syndrome is a complex genetic disorder that primarily affects the nervous system. It occurs in about 1 in 12,000 to 20,000 individuals. About causes, the syndrome is produced by a deletion in the critical region 15q11.2–q13 (60–75% of cases), paternal uniparental disomy (2–5%), imprinting defect (2–5%), and ubiquitin-protein ligase E3A (UBE3A) gene mutation (10%) [56]. Clinically, it is characterized by severely delayed development, intellectual disability, scoliosis, microcephaly, seizures, severe speech impairment (e.g., minimal to no use of words), and ataxia. There is a typical behavior with an apparent happy demeanor that includes frequent laughing, smiling, and excitability. Artigas-Pallarés et al. [57] showed high resistance to pain in about two-thirds of patients (67%) with Angelman syndrome. Nevertheless, the topic needs to be better established. Other studies, indeed, reported pain and discomfort with feeding [58], as well as visceral pain [59]. In non-communicating intellectual disability or severe cognitive disability, pain is very difficult to assess. Thus, it is often a challenge to establish if there is a nociception issue or a difficulty in the self-report of symptoms [60].

##### Prader Willy Syndrome

Prader Willy syndrome, is a congenital multisystem disease with considerable clinical variability. It is due to different genetic mechanisms that lead to the absence of the expression of the paternal genes located in the region 15q11.2–q13 for deletion (about 60%) or maternal uniparental disomy of chromosome 15 or both 15s from the mother. Prader Willy syndrome is a rare disease whose prevalence is between 1:15,000 and 1:25,000 [61]. About clinical features, the trend is typically biphasic. The neonatal period and early childhood are characterized by marked muscular hypotonia, and the delayed acquisition of the main stages of psychomotor development. Subsequently, between the second and fourth years of life, there is a progressive improvement of the hypotonia and the appearance of worsening hyperphagia. In a few years, a high degree of obesity resistant to dietary and pharmacological treatment is established, leading to serious complications of a cardiorespiratory, metabolic, and osteoarticular nature. Notably, it is the most common cause of genetically based syndromic obesity. Endocrine dysfunctions, such as cryptorchidism and hypogonadism/hypogenitalism, central hypothyroidism, growth hormone deficiency, central hypoadrenalism, and osteoporosis/osteopenia are added. Moreover, intellectual disability, behavioral and psychiatric disorders (e.g., self-harm, depression, anxiety, sleep disorders, autism spectrum disorders, ADHD, obsessive-compulsive disorder, psychosis) are described.

Among the clinical characteristics, there is a tendency to self-injury and reduced sensitivity to painful stimuli [62]. In their neurophysiological investigations, Priano et al. [63]. demonstrated that these findings can be related to a DRG involvement inducing high pain threshold.

##### Chromosome 15q Duplication Syndrome

It is a rare genetic condition due to one extra maternally derived copy of the Prader–Willi/Angelman critical region (PWACR), a region approximately 5 Mb long within chromosome 15 (15q11.2-q13.1). The severity of the condition and the associated signs and symptoms vary based on the size and location of the duplication and which genes are involved. Common features include developmental delay, intellectual disability, autism spectrum disorder, hypotonia, seizures (e.g., infantile spasms), scoliosis, slow growth, communication difficulties, behavioral problems (e.g., self-injurious behaviors), and distinctive facial features (high and/or cleft palate). In an online survey conducted in 840 families with children with the disease, a high pain threshold was reported in 87% of children. This condition was mostly observed in the isodicentric variant (85.6%) [64].

##### Chromosome 4 Interstitial Deletion

A case of pain insensitivity was described in a 12-year-old girl with a de novo interstitial deletion at the long arm of chromosome 4 between band q31.3 and q32.2 (Cr4 del q31.3 to q32.2). The deletion included the TECPR2 gene (14q32.31). The clinical picture showed developmental delay, multiple congenital abnormalities, and moderate intellectual disability (IQ 47). In history, there was no evidence of pain perception to injuries, which normally evoke pain and, in turn, absent grimacing or crying after falls, cuts, or bruises. It induced severe disability caused by painless injuries and bone fractures. The neurological examination manifested a high pain threshold with preserved tactile sensitivity [65].

## 4. Research and Perspectives

The Nav1.7 channel is a key element in the nociception pathway. In mouse acute/chronic pain models, Shields et al. [66] showed that Nav1.7 inhibitors (i.e., the acylsulfonamide compounds GX-201 and GX-585) can have an important antinociceptive activity after a single dose. Ralfinamide is a Nav and calcium channels blocker and induces sensitization of CNS neurons, through NMDA-receptor modulation. Preclinical studies on this molecule showed anti-nociceptive activity in the animal models of neuropathic pain [67], but clinical efficacy should be better proved. Based on ralfinamide, other molecules, such as the (S)-2-((3-(4-((2-fluorobenzyl) oxy) phenyl) propyl) amino) propanamide (QLS-81), were preclinically evaluated [68]. An interesting computer-aided drug discovery study yielded a new Nav1.7 inhibitor (DA-0218); it could be helpful against paclitaxel-induced CIPN [69]. The translational research must necessarily provide further details to verify the clinical efficacy and safety of these compounds.

The nociception selectivity of these potential pain killers is the key to their clinical use. Although several sodium channel-blocking anticonvulsants, such as carbamazepine and lamotrigine, are commonly used against neuropathic pain, their poor selectivity for sodium channel isoforms is a great limitation. For example, the Nav1.7 channel is expressed widely in all sensory neuron subtypes (including olfactory neurons) apart from proprioceptors. It is also found in the hypothalamus and some parenchyma, such as the pancreas [70]. On these bases, Weiss et al. [71] proved that several Nav1.7 loss-of-function mutations cause anosmia. Novel Nav1.7-targeted analgesic drugs should be highly selective for DRG terminals.

An interesting field of study is the role of modulatory analgesic pathways on the NaV1.7 signaling. The link between the Nav17-based nociceptor functioning and the opioid system falls within this scope. Pereira et al. [72] showed that Nav1.7 loss of function requires activation of μ- and δ-opioid receptors.

Gain-of-function of Nav 1.9 sodium channel subunits could also offer interesting research perspectives. As Sopacua et al. [73] suggested, several findings could be achieved by the study of small-fiber neuropathy. It is a disorder of thinly myelinated Aδ and unmyelinated C fibers due to gain-of-function variants of genes encoding for the Nav 1.9 and other channel subtypes.

The NTRK1 gene (or TrkA) encodes for the neurotrophic tyrosine kinase receptor (NTKR) family. It binds NGF [23] and its mutations can lead to HSAN-IV (or CIPA). Several pieces of research focused on the NGF/TrkA signaling as a therapeutic target for pain [74] and other aims, such as anticancer actions [75]. Research must clarify the efficacy and safety profiles of these molecules as the pathway is involved in a wide range of biological effects [23].

Furthermore, the preclinical research can develop an ad hoc animal model for investigating peripheral/central neuropathy. For instance, recently, Tamim-Yecheskel et al. [76] generated a TECPR2 knockout mouse model that exhibits behavioral pathologies observed in HSAN-IX. Model features include sensory and gait defects, abnormalities in motor coordination, hypersensitivity to thermal stimuli, and peripheral/central neuronal/fibers damages. This approach can be useful for studying neurodegenerative diseases and verifying potential links between the pathways of nociception and other intracellular signaling processes, such as those regarding cancer biology [77]. In addition, it could also offer important information for the development of molecules useful for the prevention or treatment of peripheral neuropathies, such as CIPN [78].

Finally, research must verify the functioning differences between the peripheral receptors (nerve endings) and the central medullary ones about NaV1.7 channels. The explanation for the failure of peripherally targeted NaV1.7 inhibitors can be found in this difference. Interestingly, MacDonald et al. [79] demonstrated that the central NaV1.7 terminal is associated with opioid signaling. Thus, a therapeutic strategy through a combination of opioids and centrally Nav1.7-targeted agents should require a necessary in-depth study.

Several clinical investigations are ongoing to evaluate the efficacy of potential therapeutic strategies, to better define the pathophysiology, and to build datasets on clinical features of hereditary pain disorders (Table 4).

## 5. Conclusions

Although pain represents the integration of multiple elements, nociception is an essential phase in the process. This is the electrophysiological phase of the pain pathway and provides encoding and processing of noxious stimuli. Nevertheless, nociception is a very complex phenomenon with many aspects that have yet to be defined. Several alterations can affect this process. Genetic disorders featuring increased pain threshold or pain insensitivity compose a heterogeneous group that is receiving growing attention from researchers. For instance, monogenetic disorders, producing hereditary sensory and autonomic neuropathies, are characterized by reduced or absence of pain sensitivity. To date, disparate genes involved in low or absent pain perception have been identified. These genes encode for the Nav channels 1.7 and 1.9 (*SCN9A*, and *SCN11A*), *NGFβ* and its receptor TrkA, as well as the transcription factor PRDM12 and other structural proteins or signaling molecules. Furthermore, pain insensitivity was described in diverse numerical and structural chromosomal abnormalities. 

Taken together, the study of genetic disorders showing increased pain threshold integrates with pain physiology research. Both fields of study offer valuable findings useful for the development of new pain relievers. A wide range of factors need to be considered while choosing an ideal gene for pain suppression or modulation. For translational perspectives, it is essential to fully understand the physiological mechanisms of nociception and their changes, as well as the close correlations between nociception and inflammatory phenomena, especially those responsible for pain chronification. 

## Figures and Tables

**Table 1:** Main properties of sensory receptors (free nerve endings and organs) [9,10].

Type	Structure	Fiber and Diameters	Axonal Conduction Velocities	Location	Stimuli	Act. Thresh.	Effects
Free nerve endings	Minimally specialized nerve endings	C	0.5–2 m/s	Skin	Polymodal Temperature	High	Persisting pain sensations
Free nerve endings	Minimally specialized nerve endings	Aδ	3–30 m/s	Skin	Pressure Temperature	High	Rapid, sharp pain
Free nerve endings	Minimally specialized nerve endings	Aβ	6–12 m/s	Skin	Pressure	High	Rapid, sharp pain
Meissner’s corpuscles *	Encapsulated; dermal papillae of glabrous skin	Aβ 6–12 μm	—	Most common mechanoreceptors of “glabrous” skin	Touch, pressure (dynamic)	Low	Somatosensory acuity, (digital extremities and palmar skin)
Pacinian corpuscles	Encapsulated; onion-like covering	Aβ 6–12 μm	—	Skin, Subcutaneous tissues, viscera	Vibration (dynamic, 250 Hz) Deep pressure (quasi-static)	Low	Location of touch sensations
Merkel’s disks °	Encapsulated; associated with peptide- releasing cells	Aβ	—	Skin, hair follicles	Low vibrations (5–15 Hz)	Low	Information on pressure, position, and deep static touch
Ruffini’s corpuscles	Enlarged dendritic endings. Elongated capsules	Aβ 6–12 μm	—	Skin (deep layers)	Mechanical deformation. Thermoreceptors	Low	Kinesthetic control
Muscle spindles	Highly specialized (intrafusal fibers)	Ia and II	—	Muscles	Muscle length	Low	Stretch reflex
Golgi tendon organs ^	Highly specialized	Ib	—	Tendons	Muscle tension	Low	Golgi tendon reflex (sensory component)
Joint receptors	Minimally specialized	—	—	Joints	Joint excursion	Low	Limbs position and movement

Legend: * They have a role in peripheral and diabetic neuropathy. ° It is fundamental for tactile discrimination. ^ Large-diameter rapidly conducting Aα fibers and insensitive to passive muscle stretch.

**Table 2:** Monogenic disorders featuring pain insensitivity.

Disease	Gene(s)	Encoding/Inheritance	Pain Features	Ref.
HSAN Type I	SPTLC1 (HSAN-IA); SPTLC2 (HSAN-IC)	Reduced activity of serine palmitoyltransferase; Autosomal dominant	Painless injuries to the tongue and limbs	[29,30,31,32,33]
HSAN Type II	WNK1 (HSAN-IIA) KIF1A FAM134B SCN9A (HSAN-IID)	TRPV4 (WNK1 gene); Nav1.7 (loss of function of SCN9A); Kinesines (KIF1A) Autosomal recessive	Congenital insensitivity to pain. Global pain insensitivity	[34,35,36]
HSAN Type III	IKBKAP	ELP1; Autosomal recessive	Pain insensitivity or reduced pain sensitivity	[28,37]
HSAN Type IV	TRKA	Autosomal recessive	Congenital insensitivity to pain with anhidrosis (CIPA); Global pain insensitivity	[38,39,40,41]
HSAN Type V	NGFβ	NGF; Autosomal recessive	Congenital insensibility to pain, lack of temperature sensing; reduced C-fibres	[42,43]
HSAN Type VI	DST	Dystonin; Autosomal recessive	Reduced pain sensitivity	[44,45]
HSAN Type VII	SCN11A	NaV1.9 channel. Gain of function with sustained depolarization Autosomal dominant	Decreased pain sensitivity	[46,47,48,49]
HSAN Type VIII	PRDM12	PRDM12; Autosomal recessive	Non-global pain insensitivity	[50,51]
HSAN Type IX	TECPR2	Autosomal recessive	Decreased pain sensitivity	[52,53,54]

Abbreviations: HSAN, hereditary sensory and autonomic neuropathy; ELP1, elongator complex protein 1; NGF, nerve growth factor; PRDM12, PR domain containing member 12.

**Table 3:** Numeric/structural chromosomal abnormalities with reduced pain or insensitivity.

Disease	Genetics	Encoding/Inheritance/Pain Features	Ref.
Angelman syndrome	Deletion or mutation in the maternal chromosome region containing the *UBE3A* gene	High resistance to pain in about two-thirds of patients (67%)	[56,57,58,59,60]
Prader Willy syndrome	Paternal 15q11.2–q13 deletion	A high pain threshold is a very common finding	[61,62,63]
Chromosome 15q duplication syndrome	Duplication of the PWACR region (15q11.2–q13.1)	High pain threshold in 87%	[64]
Chromosome 4 interstitial deletion	Cr4 del q31.3 to q32.2	High pain threshold with preserved tactile sensitivity	[65]

Abbreviations: PWACR, Prader–Willi/Angelman critical region.

**Table 4:** Ongoing clinical investigations on hereditary sensory and autonomic neuropathies.

Disease	Study type	Intervention(s)	Outcome	Study ID *
HSAN-I	RCT	L-serine supplementation (400 mg/kg/d, 2 years)	Disease severity °	NCT01733407
HSAN-III	RCT	Proprioceptive inputs (cortical representation)	Proprioception and Sensorimotor Control	NCT02876939
HSAN-III	Observational Prospective	N/A	Database. Predictive biomarkers of disease progression and severity	NCT03920774
Rare Disease	Observational Prospective	N/A	Genetics; Pain; Diagnosis (e.g., skin biopsy)	NCT02696746
Rare Diseases ^	Observational Prospective	N/A	Patient Registry & Natural History Study	NCT01793168
Pain sensitivity alterations †	Observational Prospective	N/A	Pain score, Measures of quality of life, Neurophysiology, Imaging	NCT02696746

Legend: * From https://clinicaltrials.gov (accessed on 30 March 2022); ° Charcot Marie Tooth Neuropathy Score; ^ Coordination of Rare Diseases at Sanford Registry (CoRDS); † Painful Channelopathies Study. Abbreviations: HSAN, hereditary sensory and autonomic neuropathies.

## Data Availability

The datasets generated during and/or analyzed during the current study are available from the corresponding author on reasonable request.
